# Recumbency in the Treatment of Infantile Paralysis

**Published:** 1907-09

**Authors:** Adoniram B. Judson

**Affiliations:** New York


### Recumbency in the Treatment of Infantile Paralysis.

By ADONIRAM B. JUDSON, M.D., New York.

[*Author's Abstract.*]

IN the ever-changing treatment of disease the influence of environment is receiving unusual attention, as is seen in the management of tuberculosis of the joints. The influence of the lapse of time is also better understood. Medicines are given in small doses for very long periods, and the effects of time on the body are more clearly seen to influence the course of disease and the action of remedies.

In the treatment of infantile paralysis I propose a method which relies exclusively on the influences of environment and the the case is likely to be seen by an orthopedic surgeon. As soon as the disease is recognised I would limit the patient to the recumbent position till there is no possibility of further recession of the paralysis. The period of spontaneous recession extends over several months. During this time the difficult task must be undertaken of keeping a child, well in every other way, off his feet at an age when he should be learning to walk. In some cases eighteen months should be occupied in this way. The common belief that such a patient requires exercise, especially of the affected limbs, will give rise to criticism and objections. A simple argument will not prevail in the family circle, and the physician's word will hardly prevent the little patient from having many a romp. And when the case ends there will be differences of opinion. If some lameness results, it may be said that the patient should have had more exercise, and if there is no disability at all, after the strict observance of recumbency, it may be said that there had been very little the matter with the child.

The argument is as follows: It will be recalled that the ill effects of joint disease are seen more commonly in the lower extremities than the upper because tuberculous action is subject to resolution in the epiphyses of the shoulder, elbow and wrist, but often goes on to destruction of the articulating surfaces of the hip, knee and ankle. And when it is noted that the arms are free while the legs bear the weight of the body it is reasonably inferred that the joints of the lower extremities when affected, or even suspected, should be protected by either recumbency or appropriate apparatus. The conclusion is a plain proposition and needs no discussion or verification. It shares the simplicity of Jenner's argument when he traced the relation of cause and effect and prescribed vaccination. In another field Finlay, walking with his eyes open, apprehended the relation of cause and effect and prescribed the sequestration of the mosquito.

The necessity of reforming the environment of the lower extremities having been derived from clinical observations of joint disease, can practical conclusions be drawn in a similar manner from observing the course of infantile paralysis? Disability from this disease is seen eight times as often in the lower as in the upper extremities, and yet in the early stage the paralysis is found in all parts of the motor nervous system. The muscles of the recumbent patient are in very moderate use and in a position entirely favorable to spontaneous recession of the paralysis. The arms and hands retain this advantage when the patient is erect, but the impaired muscles in the legs and feet give way at once when they meet the resistance of the weight of the body. They rapidly become elongated and attenuated, and could not well be placed in an attitude more destructive of the possibility of restoration.

When prescribed recumbency shall give to all parts the same environment, recession of paralysis will be equally encouraged in the lower and upper limbs, the disproportion of eight to one will disappear, and the sum of deformity from this disease will be materially reduced.

The value of the method is thus *proved,* but it is not readily *demonstrated.* When comparing methods it is not easy to show that one is better than another. It may always be said that a case cited in behalf of a certain method may have been one that would have done well under any treatment. Tables of carefully recorded cases might lead to correct estimates, but studies of this kind are difficult and have not escaped criticism. Dr. Gaillard Thomas said with wit and wisdom that if there is anything more misleading than facts it is figures. Medicine and surgery are still outside of the realm of exact science. Therefore we welcome every logical and reasonable resource of prevention and treatment.

Passive motion, resistance exercises, electricity, massage, local applications and judicious medication should be continued. They cannot interfere with the treatment proposed, and their observance may make it easier persistently to maintain recumbency, the

53 Washington Square.

